# Integration and Typologies of Vulnerability to Climate Change: A Case Study from Australian Wheat Sheep Zones

**DOI:** 10.1038/srep33744

**Published:** 2016-09-27

**Authors:** Jianjun Huai

**Affiliations:** 1 Department of Economics, College of Economics and Management, Northwest A&F University, Yangling, Shaanxi, China

## Abstract

Although the integrated indicator methods have become popular for assessing vulnerability to climate change, their proliferation has introduced a confusing array of scales and indicators that cause a science-policy gap. I argue for a clear adaptation pathway in an “integrative typology” of regional vulnerability that matches appropriate scales, optimal measurements and adaptive strategies in a six-dimensional and multi-level analysis framework of integration and typology inspired by the “5W1H” questions: “Who is concerned about how to adapt to the vulnerability of what to what in some place (where) at some time (when)?” Using the case of the vulnerability of wheat, barley and oats to drought in Australian wheat sheep zones during 1978–1999, I answer the “5W1H” questions through establishing the “six typologies” framework. I then optimize the measurement of vulnerability through contrasting twelve kinds of vulnerability scores with the divergence of crops yields from their regional mean. Through identifying the socioeconomic constraints, I propose seven generic types of crop-drought vulnerability and local adaptive strategy. Our results illustrate that the process of assessing vulnerability and selecting adaptations can be enhanced using a combination of integration, optimization and typology, which emphasize dynamic transitions and transformations between integration and typology.

Climate change can have a huge impact on socioeconomic and ecological systems^1^. Through recent decades, interdisciplinary research has contributed to improving our knowledge of empirical methodologies related to assessing climate change vulnerability^2^,^3^,^4^. In recent years, integrated indicators combining climatic and non-climatic elements at different scales have been developed for quantifying vulnerability^5^. These aim to develop robust and credible measures incorporating diverse methods such as principal components analysis (PCA)^6^ and draw on research into adaptive mechanisms^7^. For example, vulnerability is considered to be a function of exposure, sensitivity and adaptive capability^8^ or it can be calculated by different formulations in different contexts^9^,^10^.

However, the current generation of integrated methods and diverse calculation techniques can lead to confusion in selecting scales, indicators and calculation methods. Existing integrated indicator methods such as integrated assessment (IA) and ‘Drivers-Pressure-State-Impact-Response’ (DPSIR)^11^ are not well suited for combining multiple spatial, temporal scales and socioeconomic-ecological dimensions in a locality due to limited knowledge of several factors, such as the availability of a farm’s perceived adaptations. This has led some scholars to question what indicators can accomplish in the domain of climate change vulnerability^12^ because some indicators represent neither what researchers want to measure nor allow for the effect of feed-back^13^. These integrated indices also ignore the interactional effects of complementarities and substitution between indicators in PCA, which can misinform stakeholders such as farmers^14^,^15^ and result in a science-policy gap^16^. Uncertainties combine with different methodologies and diverse place and time scales, due to the different contexts. Local socioeconomic conditions and wider ecological environments vary widely. Much of the relevant measurement and methodology for understanding vulnerability and adaptation to climate change can therefore only be appreciated in a particular context.

A typology can help to identify specific types of vulnerability, adaptations, mitigation and stakeholders, to generate a general framework for understanding interactions between environmental stress and human activities^17^. For instance, typologies of crop-drought vulnerability distinguish between resilient and sensitive cases and their different adaptations according to their dissimilar socioeconomic conditions^18^. Also, local income, household size and climate give rise to a spatial typology of human settlement in research that rejects a one-size-fits-all adaptive decision in England^19^. Typologies have been used to appreciate environmental heterogeneity, assess overarching adaptive activities, analyze the cost of climatic adaptation and mitigation; and to consider the needs of all types of primary producers^20^,^21^,^22^.

In order to reflect heterogeneity on different dimensions, constructing a typology involves a series of questions. It is important to identify the special conditions of local climates at different scales^23^, the regional limits of adaptive capacity^24^ and to consider the interests of stakeholders^25^. The monthly growing response of vegetation to drought in some regions is selected to provide some useful information concerning the operations of the time scales^26^. Under the framework of “Loss-response”, the time dimension includes before, during and after disaster periods, the spatial dimension contains community, town, country and province, while there are also economic, institutional, social, and environmental attributes^27^. A typology comprised of six content themes: *study region, climate hazards, relevant sector, impacts of concern, potential adaptation options, decision processes and tools for adaptation*^7^. The assessment of vulnerability should answer the “4W2H” questions: *What are the goals? How is the assessment of vulnerability framed? What are the technical methods? Who participates in the assessment? How will it be used to facilitate change?*^28^ Thereby a typology can identify different types of temporal and spatial scales, attribution, research objectives, adaptations, methods of measurement and policy tools. Nevertheless, few researchers to date have systematically described the process of decision-making in climate change adaptation and none has clarified the relationships between typology and integration or emphasized the match and optimization of indicators.

In the paper, I test the hypothesis that the process of assessing vulnerability and selecting adaptations can be enhanced by using a combination of integration, optimization and typology. I apply a top-down method to develop a six-dimensional analysis framework that considers location, time, people, focus, method and adaptation to answer the “5W1H” questions: “*Who (some people) are concerned about how to adapt to the vulnerability of what to what in some place (where) at some time (when)*?” It includes the following, more specific questions:

(1) *Where does climate change occur? (Where)*

(2) *When are people affected by climate change? (When)*

(3) *Who is concerned about climate change? (Who)*

(4) *Which systems are affected by climate change? (Of What)*

(5) *Which kinds of climate change occur? (To What)*

(6) *How will the people assess and adapt to climate change? (How)*

The paper explores an integrative understanding of vulnerability and adaptation pathways through integrating socioeconomic indicators into the measurement of vulnerability, comparing different ways of calculating vulnerability, classifying resilient and sensitive cases and constructing a typology of adaptation in a defined system based on empirical data. Drought data was obtained from recent research^29^ while the rest of the data comes from the Australian Agricultural and Grazing Industries Survey (AAGIS).

I develop a systematic method for integrating data at appropriate scales, optimizing the calculation of vulnerability and constructing a typology of adaptations. I contribute to the literature on integration, optimization and typology of vulnerability and adaptation using the case of Australian crop-drought vulnerability. Specially, our novelties are (1) a six-level analysis framework for transforming typology to integration, (2) a detailed seven-fold typology of vulnerability and adaptive strategies for Australian crops, (3) and a dynamic transition method for transformations between integration and typology.

## Six-dimensional analytical framework

**Figure 1:**
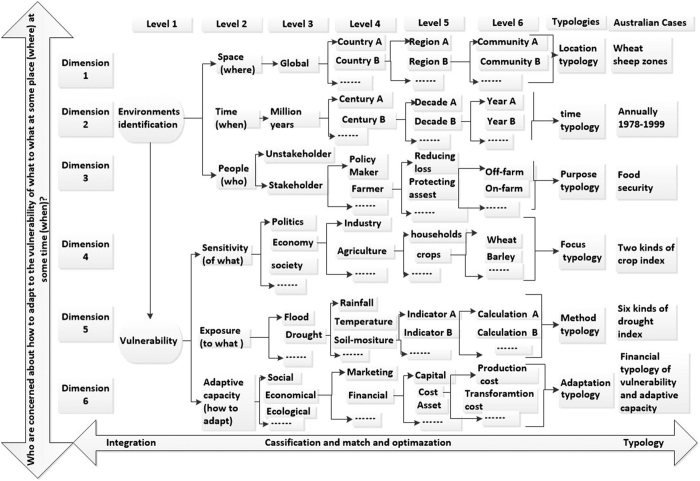
Classifying vulnerability is addressed by answering the “5W1H” questions: Who (some people) are concerned about how to adapt to the vulnerability of what to what at some place (where) at some time (when)?

In a defined system, *who* (some people) would be concerned about *how* to adapt to the vulnerability of *what* to *what* in some place (*where*) at some time (*when*)? To answer the “5W1H” questions, the analysis framework of vulnerability should contain six typologies of space, time, stakeholders, focus, method, and adaptation (Fig. 1).

The first three steps of systematically defining environments are: ascertaining the types of purposes which are significant for stakeholders, identifying location types to define the study areas at appropriate spatial scales and selecting the time scale of the research. Careful selection of areas to investigate according to spatial heterogeneity in socioeconomic and ecological systems can answer the question “*Where does climate change occur?*” A typology of locations meansthat the researcher can locate an appropriate spatial scale to assess vulnerability at global, national, regional and community levels through scientific methods. Climate change has different characteristics and influences during different time-periods, therefore it is important to answer the question “*when are the people affected by climate change?*” A time typology implies that the researcher should find the effective temporal scale from millennia, centuries, decades and years, according to the frequency and severity of climate shocks. Due to the heterogeneity of regions, different groups of people have dissimilar responses to climate change^30^. For example, farmers who have experienced a severe drought will be more concerned about climate change in the future than those who have never known drought conditions. A typology of purposes makes it possible to identify “*who is concerned about climate change?*” based on their different experiences of it. In general, climate change at different spatial and temporal scales affects people in diverse ways, so I need to select appropriate typologies to define all relevant systems.

The next three steps include identifying what I call “focus types” to measure the sensitivity “of what”, selecting the types of method to assess the level of exposure to climate shocks and finding types of adaptation to reduce the vulnerability at practical dimensions. Within a defined system, many kinds of climatic stocks, such as droughts, floods, freezing conditions and hail storms, etc. may happen simultaneously and affect various kinds of activity. The focus type shows paths for exploration to select “of what to what” to answer “*which kinds of climate change occur?*”and “*which systems are affected by climate change?*” This may consider the vulnerability of crops to drought, the vulnerability of rural livelihoods to water scarcity etc. The “method typology” provides the processes needed to measure vulnerability, such as conceptualizing and calculating crop-drought vulnerability. The question of “*how will people assess and adapt to climate change?*” involves assessing vulnerability and selecting effective adaptations. The “adaptation typology” can provide a useful tool to assess local adaptive capacity and to select local adaptations according to regional system constraints.

The “six typologies” framework describes how to answer to the “5W1H” questions in principle. How it operates in practice can be better understood when I translate this framework into a practical methodology and apply it in a case study in Australia.

## Methodology: detailed application of the framework in Australia

### Defining the study space, time and people to answer the “where”, “when” and “who” questions

#### Identifying a typology of locations to answer the “Where” question

Australia is the world’s sixth largest exporter of aggregated food production and thus contributes significantly to world food supply. Among many broad acre agricultural production systems in Australia, wheat sheep zones are found to account for 90–95% of Australia’s crop outputs, but often suffer from long-term droughts^31^,^32^. Therefore, I select 12 wheat sheep zones as our study areas, after continually shrinking the spatial scales from country to regions, then to the main crop production regions of Australia. The wheat sheep zones cover the north and east Wheat Belt (522), the central and south wheat belt (521) in Western Australia, the Eyre Peninsula (421), the Murray Lands and Yorke Peninsula (422) in South Australia, the Mallee (221), the Willera (222), the Central North (223) in Victoria, the Riverina (123), the Central West (122), the North west Slopes and Plains (121) in New South Wales, the Eastern Darling Downs (321) and the Darling Downs and Central Highlands in Queensland (the number in the parentheses represents the region code).

#### Discovering a time typology to answer the “When” question

I select an annual scale rather than a monthly or seasonal one because I can then use the annual socioeconomic indicators and the crops grow to harvest once a year. I select 1978–1999 as our observed period because recent literature has analyzed the vulnerability of crops to drought during recent decades but before 2000 there had been little attempt to integrate socioeconomic indicators into climate change research in Australia^28^,^33^.

#### Identifying a typology of purpose to answer the “Who” question

Many related groups of people, such as local policy-makers, scientists and farmers, etc. emerge as being relevant for research into the impact of climate change. During 1978–1999, since climate change, such as drought directly affects agricultural systems including crops and farmers, and results in huge losses of crop harvests, farmers are concerned about the vulnerability of crops. However, local policy-makers and scientists have little response to the influence of Australia’s drought on socioeconomic variations^34^. Australian farmers are most affected directly by climate change and are most concerned about its adverse impact. Therefore, I focus on the farmers as the most directly affected people.

### Measuring exposure, sensitivity and vulnerability to answer the “of what”,“to what” and “how to adapt” questions

#### Discovering “focus types” to answer the “of what” questions

The focuses types result from the process of selecting the sensitivity of something from everything according to particular conditions. Sensitivity reflects the response of a given system to climatic variation, may be influenced by socioeconomic and ecological conditions^35^. Practically everything is affected by the climate in the research fields, I am considering so that I hardly need to list and select which is their first priority. In Australian wheat sheep zones during 1978–1999, crop yields were directly affected and different crops respond differently to climatic variations^36^. Therefore, I applied the crop yield anomaly of wheat, oats and barley as a proxy for crop sensitivity to drought.

The selection of appropriate indicators and methods of calculation are key components in the empirical analysis. The “method types” include such selections and comparisons between different methods. Many researchers have used the detrended yield to assess the crop vulnerability to climate change. For example, the modeling crop yield^37^, or simulated yield at large scales can used to monitor or forecast regional variability in crop production^38^. To eliminate non-climatic effects on yields, the detrended yield was obtained by subtracting the trend yield from the actual yield^39^. The 21-year linear sliding average method was applied to remove trends in yields, while winter wheat yield was divided into trending yield and meteorological yield, which was further processed as the relative meteorological yield^40^,^41^. Following previous research^31^,^42^,^43^,^44^, I use the detrended yield and averages of actual yield to measure crop sensitivity to drought. I detrended the annual crop yield via an auto-regression function with 3-year lags. Next,I calculated two Crop Failure Index (CFI) using the detrended yield (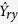) and the average yield (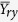), respectively,as in equation (1). A CFI of more than one indicates crop failure and the greater the CFI, the more severe is the crop failure.


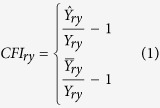


#### Identifying “method types” to answer the “to what” questions

Drought is a major problem in Australia^45^; the Millennium droughts for example reduced aggregate agricultural production and exports which resulted in global fluctuations in food prices^46^. Recent droughts, especially in 2007, also caused significant destruction^47^. Exposure is the magnitude and frequency of extreme climate events^48^, so I selected drought as an obvious exposure element of climate change according to the focus types.

According to the method types, the selected indicators of drought include rainfall, temperature and soil-moisture, etc. Due to its direct link with the planting of crops, I used soil-moisture to estimate the severity of drought. Then, I compared the results using different calculation methods of the drought index using soil-moisture data. Many agrohydrologists have researched the conceptual and practical issues of scales and scaling, for instance, why scaling problems arise, the defineand types of scales and the key questions with regard to upscaling and downscaling were discussed^49^. The differences in the spatiotemporal patterns of temperature and precipitation are statistically significant, and the temporal trends and spatial structures of each meteorological element were not equally modified^50^. Examining habitat loss and habitat fragmentation across different time periods and at different spatial scales is essential for understanding their joint and individual effects on plant community composition^51^. Therefore, matches between spatial scales and time scales are critical in comparing different calculation methods, which include the transformation form one spatial scale to another and a lagged offset measure of time^52^.

Comparing many different ways of performing the calculation of drought index, I selected six ways to express the drought index (DI) following the relevant literature that use at least three different calculation methods^18^,^53^,^54^,^55^,^56^. Firstly, the Soil Moisture Deciles-based Drought Index (SMDDI) of the Australian Bureau of Agricultural and Resource Economics and sciences Regions (ABARER) was used as the drought index. This is abbreviated to SNR from SMDDI in the Natural Resource Management Regions (NRMR)^29^. Map grids divide the Earth’s surface into a uniform array and since there are different planted areas in each grid, I weighted the SMDDI in each geographical grid by the percentage of its planting area in the whole research area to produce a weighted SNR, WSNR. Here, following previous literature^29^, I defined vulnerable cases to be those with a SNR or WSNR greater than 0.03. Secondly, I indirectly measured droughts using SNRM or WSNRM, which are defined as the ratio of the SNR or WSNR to its own mean. I ignored SNRM or WSNRM when it was zerobecause this simply indicates there was no drought. MSNR or MWSNR is the reciprocal of the SNRM or WSNRM, respectively and was used as a wetness index. Finally, I ascertained the appropriate research duration through comparing the correlations between these six types of drought indexand annual yield as well as failure for three crops in the same and offset (lagged by one) year. All calculation methods are defined in equation (2):


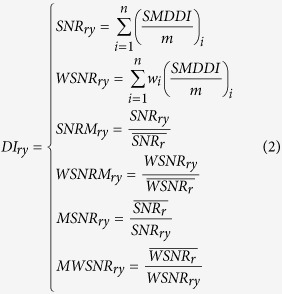


where rindicates the type of region and *y* stands for the year, *m* is the number of grids in each NRMR, *i* is the number of grids and 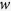 is the weight or percentage of the grid in each ABARER.

#### Building the “adaptation type” to answer the “How” questions through classification, integrating, optimizing and typology

Sensitivity as “the degree to which a system is affected, either adversely or beneficially, by climate-related stimuli”^57^, while resilience refers to the magnitude of disturbance that can be absorbed before a system changes to a radically different state as well as the capacity to self-organize and the capacity for adaptation to emerging circumstances^58^,^59^. Thus, sensitivity and resilience have common elements of interest: the shocks and stresses experienced by the social-ecological system, the response of the system, and the capacity for adaptive action^3^. Given certain exposure, sensitivity maybe negative with resilience. Here, I only classify resilient and sensitive cases, which means the resilience equals to less sensitivity so that I can identify some vulnerable cases.

First is to classify resilient and sensitive cases according to the ranks of CFI and DI. The relationships between sensitivity and exposure determine that the method types should compare and integrate the CFI and DI into vulnerability at appropriate space and time scales. I classified the resilient and sensitive cases and the financial typology of vulnerability. After ranking the CFI and DI, I considered “resilient” cases if the CFI was below the median while the DI was above the median. “Sensitive” cases were defined to be those where the CFI was above the median and the DI was below the median.

Second is to integrate and optimize vulnerability. Although exposure and sensitivity are integrated into vulnerability through various calculations by different researchers, to simplify the question, I defined the crop-drought vulnerability index (VI) as sensitivity divided by exposure and then used the divergence of yield from its own mean (DY) as the baseline of vulnerability. Since DY reflects the variance of the actual yield loss relative to the average, different levels of VI representthe variance of relative crop yield loss to the variance of relative drought, thereby I selected the optimal crop-drought vulnerability index as the smallest 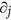 that is the difference between theVI and the DY^9^,^10^.


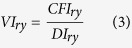



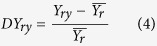



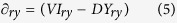


Selecting and integrating adaptive capacity is the third step. Adaptive capacity reflects the ability of a system to adjust to changing climate in a way that reduces potential damage and takes advantage of any associated opportunities^60^. The socioeconomic attribution of adaptive capacity determines various method types; the key operational measurements of adaptive capacity is about how to identify which indicators from all possible socioeconomic indicators using first-classifying-then-integrating methods. I investigated what parameters indicate a statistically significant difference between resilient and sensitive cases using 146 socio-economic indicators from the AAGIS. Then, I took the indicators from the 1^st^ principal component (PC1) from a PCA of the selected indicators. I identified significant indicators through a Spearman’s correlation test among the indicators included in *PC1* and levels of vulnerability for resilient and sensitive cases, respectively. Finally, I characterized regional adaptive capacity using the parameters of capital, cost, debt, receipts, farm equity, family farm, farm performance and other assets.

Providing typologies of vulnerability and adaptations based on regional financial constraints is the fourth step. The setting up of reasonable criteria is a critical step in defining the adaptation and vulnerability types. In systems affected by climate change, social heterogeneity for different stakeholders at particular locations and times are relevant for such criteria. Farmers in Australian wheat sheep zones, in the period 1978–1999, focus on their financial costs and benefits from climate change. To find financial constraints for every region, I summed all the significant indicators for each category. Total opening capital and debt presents one category; total receipts, farm performance, family performance and assets are the sum of their indicators respectively. Total cost equals the sum of all cost indicators except expenditures on fodder, fertilizer and crop & pasture chemicals. Lack of capital, low receipts and assets, family farm and farm performance, as well as higher costs and debt are the regional constraints to adaption to climate change.

## Results: effectiveness of the “six typologies”

### Method typology through optimization can provide the optimal crop-drought vulnerability index

*VI*^*11*^and *VI*^*12*^ demonstrated that our comparisons and optimizations in our methodology types are helpful in measuring vulnerability. For wheat, the smallest values of *VI*^*11*^ and *VI*^*12*^ become the optimal wheat-drought vulnerability indices in each region. Similarly, *VI*^*11*^or *VI*^*12*^ for barley and oats are the optimal measurements.

### A typology of locations is beneficial for identifying local financial constraints and the spatial typology of vulnerability

#### Local financial constraints provide the criterion of a typology of locations

**Figure 2:**
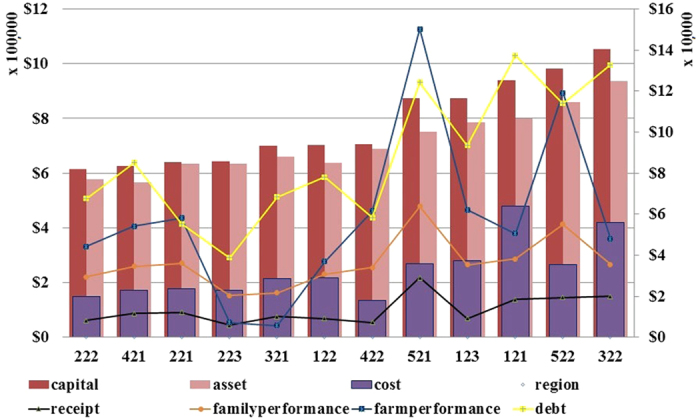
Different regions suffered from different capital constraints. The numbers under the horizontal axis represent region codes.

Figure 2 shows how each region is limited by financial factors, which effectively reflect the suitable criterion to express local heterogeneity. For instance, Mallee (221) is a capital-constrained region, which is also limited by having fewer assets. North West Slopes and Plains (321) are limited by greater agricultural costs while lower farm receipts have a negative influence in Central West (122). Central North (223) is restricted by farm performance and family farm, while greater total debts occur in Central and South Wheat Belt (521).

The methodology type with regional heterogeneity successfully matches the typologies of regions and their constraints. Table 1 shows how the resilient and sensitive regions for different crops are affected by different financial conditions. The resilient and sensitive cases have diverse financial characteristics. The more sensitive regions tend to have less capital, crop receipts, cost, debt, agricultural assets, and family and farm performance. For either *VI*^*11*^ or *VI*^*12*^, all influences significantly and negatively affect vulnerability. *VI*^*11*^ for wheat, barley and oats is affected mainly by costs and debts, while *VI*^*12*^ for wheat has additional influences including most of the indicators for capital, costs and debts.

#### Spatial typology of crop-drought vulnerability

**Figure 3:**
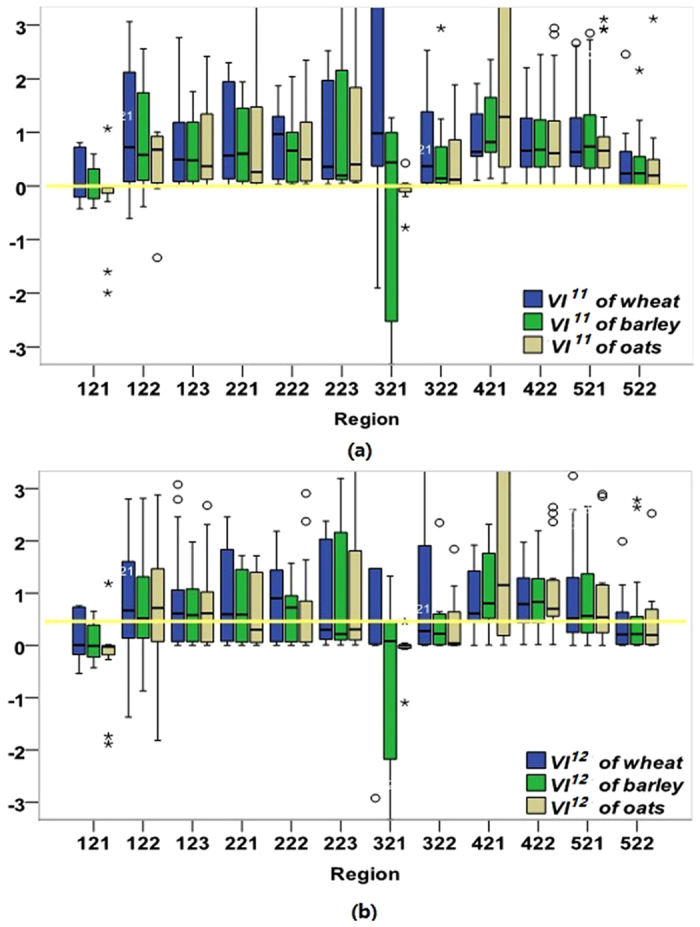
Box plots of the optimal vulnerability (e.g., VI^11^ and VI^12^) for wheat, barley, oats, and their resilient and sensitive regions. The numbers under the horizontal axis present region codes.

Further, the methodology type also clearly shows the distributions in spatial typologies of vulnerability. In Fig. 3, the box plots illustrate the distribution of sensitive and resilient regions for wheat, barley and oats in the wheat sheep zones. The longer boxes indicate a higher sensitivity while the shorter boxes indicate more resilience. For instance, wheat is resilient to drought in the North and East Wheat Belt (522), whereas it is sensitive in the Central West area (122). North West Slopes and Plains (121) is one of the most resilient regions while Central West (223) is one of the most sensitive areas for barley. Similarly, the regions of high resilience for oats include North West Slopes and Plains (121), while sensitive regions for oats include Male (221), Central North (223), and Eyre Peninsula (421).

### Time typology benefits the matching of one-year-lagged drought to crop yields

The time typology involves not only the selection of appropriate time scales, but also in finding an effective offset where appropriate. In Table 2, SNR and WSNR have a negative effect on crop yields and a positive effect on crop failure. All drought indices have significantly larger coefficients with actual crop yields and crop failures when lagged by one year compared to the same year. Therefore, I selected the drought index lagged by one year (1980–1998) as the dependent variable to investigate crop-drought vulnerability from 1981–1999.

### A typology of purposes describes the interaction between policy options and research

Once I take into consideration financial constraints, the Australian main crop and the lagged-one-year effect of the drought on the crops, not only farmers, but also the policy-makers and agricultural scientists come to a better understanding of the impact of climate change on the crops. Scientists have better opportunities to assess crop-drought vulnerability and to make useful suggestions to the government and farmers, who consequently may gain more financial support from the local economy. When policy-makers are aware of the practical issues of farmers and the scientific assessments of researchers, they are in a better position to find solutions to mitigate and adapt to the adverse impacts of climate change on farmers and crops. I can see that cooperation and coordination between farmers, scientists and policy-makers is mutually advantageous. Farmers provide survey data to scientists who can analyze this to obtain useful recommendations, which in turn helps officials design and implement effective policies to reduce the farms’ loss due to climate change.

### Method typologies benefit for the seven types of crop-drought vulnerability

In the light of the financial constraints, I can conceptualize seven generic types of crop-drought vulnerability (Table 3). Type 1 is constrained by capital in Murray Lands and Yorke Peninsula (422) for resilient wheat, in Mallee (221) and Central North (223) for three sensitive crops. Type 2, the farm asset-constrained vulnerability occurs in similar cases to the capital-constrained vulnerability. Type 3 is the vulnerability for three sensitive crops limited by total receipts in the Central West and Central North (121). The vulnerability limited by farm performance is Type 4 and it occurs for resilient oats in Eastern Darling Downs (321) and for three sensitive crops in Central West (122), Central North (223), and Eastern Darling Downs (321). For Type 5, the distributions of vulnerability constrained by family farm are similar to those of Type 4. The cost-induced vulnerability that is Type 6 occurs for resilient barley and oats in North West Slopes and Plains (121). Debt-induced vulnerability is represented in three resilient crops in some regions such as Darling Downs and Central Highlands in Queensland (322), where debt and interest payments become too onerous to be sustained by agricultural income. These typologies obtained from matching adaptive capacity and vulnerability effectively integrate space, time, crops, farmers’ financial activities and climate change together, which imply the function of adaptive capacity.

## Discussion: advantages of the “six typologies” frameworks

The “six typologies” frameworks can represent the heterogeneities of time, space and method as well as dynamic transitions and transformations between integration and typology in the assessments of vulnerability. They also provide seven useful generic types of adaptations. Although such frameworks have these advantages, they also have some disadvantages such as the various combinations of different types, the complex relationships between elements among different types and the different ways to calculate vulnerability, which will lead us to future research.

The “six typologies” framework emphasizes six heterogeneities. The environmental heterogeneity of the observed regions has shown that regional climatic variation and climate change, soil conditions and crop management are different at different dimensions and levels^61^. In addition, socioeconomic heterogeneity is worthy of more attention by “5W1H”. Seven financial categories affect all resilient and sensitive cases for the three crops, which determine the financial heterogeneity of the vulnerabilities for barley, wheat and oats in Australia. I confirmed that (i) the level of capital and receipts reduce the vulnerability for wheat; farm performance decreases the vulnerability for oats, while family farm is negatively related to vulnerability for oats. Commonly, agricultural capital investments and higher crop receipts improve the returns of farming and reduce vulnerability^62^. Here, just wheat, barley and oats are affected by differential financial capital arrangements that show the heterogeneity of crops. Droughts can cause family farms to lose income diversity and increase their debt^63^, potentially leading to a “cycle of poverty”^64^ which can affect the inter-generational succession of family farms^65^. (ii) Cost and debt add to the vulnerability for wheat and barley. Drought imposes substantial costs for entities, public ventures, commercial organizations and governments^66^. Farmers may bear increased costs due to water scarcity or the necessity of irrigation during long-term severe droughts^67^. Benmelech and Dvir^68^ demonstrated that short-term debt increases vulnerability. Our results support the idea that regional heterogeneity arises from environmental factors (e.g., local crops and climate change) and social factors (e.g., financial constraints), so creating a typology is useful for finding differences in each defined system. In total, the framework of “six typologies” reflects the heterogeneity of space, time, human factors, exposure, sensitivity and vulnerability.

Seven generic types of adaptations are valuable for making policy. I also construct seven generic types corresponding to adaptive strategies for each type of vulnerability in Australian wheat sheep zones. For Type 1 vulnerability, making additions to plant & equipment would upgrade agricultural technology. Planting more drought-tolerant varieties of crop would make more effective use of capital to help buffer harvests against drought. Alternatively, accumulating and utilizing farm equity to augment capital investment on the farm and using farm related liquid assets more efficiency may reduce Type 2 vulnerability. The methods of reducing Type 3 vulnerability may include measures to keep agricultural prices stable and protecting agricultural receipts to avoid the huge transition from farm to off-farm industrial activities. Increasing farm cash income and profit through the market mechanism or government allowance can reduce Type 4 vulnerability^69^. For Type 5 vulnerability, increasing receipts and farm income through reorganizing the management style are potentially beneficial measures for increasing the revenues of family farms^70^,^71^. Deploying technological investments and improving special management skills may also be key strategies to reduce this vulnerability that could also involve reducing costs in agricultural processes such as planting, fodder, spraying, and fertilizer, which would be a good way to reduce Type 6 vulnerability^72^. One way to reduce debt-induced vulnerability is to supervise the risk of debt through developing financial tools and reducing land purchasing^73^ (Table 3).

This typology of adaptations is intended to match the identified regions that are constrained by financial conditions with the resilient or sensitive regions for different crops. For example, Murray Lands and Yorke Peninsula (422) is a resilient region for wheat, while also being a capital and farm asset-constrained region. The match of both means capital and farm assets constrains the adaptive capacity for wheat there. Additionally, the resilient barley in Darling Downs and Central Highlands (322) is limited by cost because of the match of resilient typology of barley and the constraints of heterogeneous costs. This match is useful for creating and selecting the typology of vulnerability and adaptations.

Such a primary understanding is essential for a range of applications. First, the typology of adaptation provides a device that can help inform stakeholders as to what type of vulnerability they face and to adjust their adaptive strategies accordingly. Second, the typology puts forward a way that human factors can be better incorporated into crop–climate models, improving our appreciation of how underlying socioeconomic processes affect crop yields and showing where they are vulnerable to these changes. Third, the typology is still useful for those regions listed in Table 3, such as Eyre Peninsula where there is no match between limited regions and identified regions. If the resilient or sensitive regions have not been constrained by any of the listed categories in Fig. 3, I should consider more socioeconomic indicators beyond our list, such as human capital and crop prices, which give the policy-maker new guidelines and lead us to potential further work. If the regions exclude the resilient or sensitive cases where I know their socioeconomic features, there are at least two categories: the good crop harvest regions in drought conditions and the crop failure regions without drought, which require us to further rebuild the typology of the vulnerability and adaptation.

There are dynamic transitions and transformations between integration and typology. I construct and apply a plane grid that comprises six dimensions and six levels (see Fig. 1) to express integration and typology. This scheme is intended to assist with the common need for robust decision making within the process of assessing vulnerability and applying appropriate adaptation measures. I develop and test the new “adaptation pathways” through providing a linear sequence, and for instance, identify farmers’ options for actions for reducing farm losses due to adverse climatic events, from more general to more specific using decomposition methods. Meanwhile, I define and match what the farms are concerned about such as, which crops, which climate elements, how to assess and respond to shocks, etc. in the vertical direction using inductive methods. The combinations of the inductive and deductive methods here means I have to match and optimize the integration and typologies in these six dimensions and levels when answering the questions of “5W1H”^42^.

**Figure 4:**
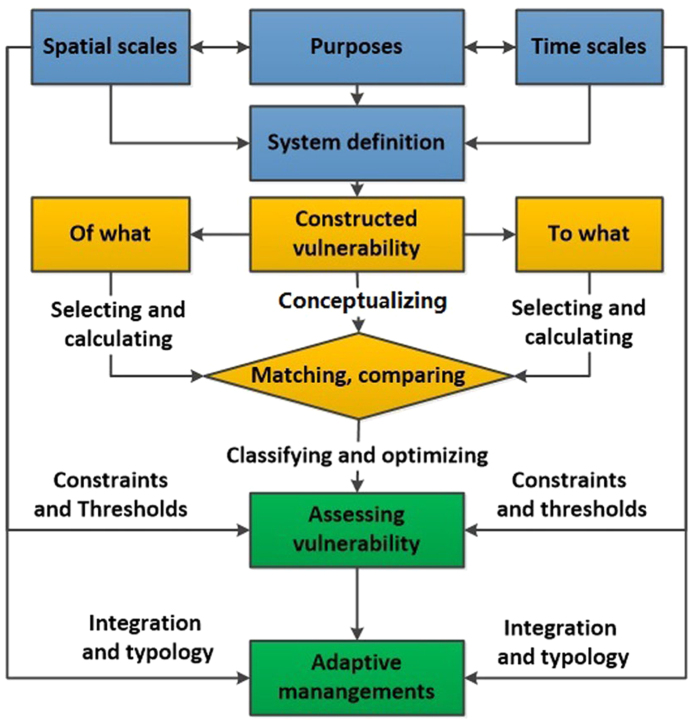
General steps to use the framework of “six typologies” in the vulnerability and adaptation management.

From the point of view of the user, I provide a set of general practical analysis steps (Fig. 4) through demonstrating the methodology of the Australian case. First, define the research systems through selecting and matching the most appropriate space, time scales, people and purposes. For example, I select farmers concerns about climate change in Australian wheat sheep zones in 1978–1999. Then conceptualizing, comparing and matching “vulnerability of what to what” is the key to managing vulnerability, among which exposure and sensitivity are used to select appropriate indicators and explore various calculation methods to optimize the measurement of vulnerability. For example, I explored crop-drought vulnerability through matching two kinds of CFI with six kinds of DI. The third is to assess vulnerability through classifying the cases according to their constraints and thresholds at appropriate spatial and temporal scales. Classifications of resilient and sensitive cases in the context of local limitations were also developed in the study. Finally, a typology of adaptations was derived according to the typology of vulnerability. This match, expressed in Table 3, helps us to clearly identify local adaptations according to local heterogeneity.

The two differences of scale-selections and lagged effects between the current study and its predecessors^26^ further can show the usefulness of our methodology. It focused on the activity and process at monthly scale without considering human adaptation, and acknowledged that such lag effects existed but usually become short demonstrated by the response of vegetation activity, forest growth, and the ANPP to short drought^26^. In Fig. 1, six dimensions and six levels mean that we can select different time scales with the same crop in different spatial scales. The integration, matching and optimization as well as typology should be affected by the type of scientific question, the availability of data and the matches of different spatial and temporal scales in the assessments of vulnerability. Here I selected vulnerability of annual crop yield to drought in wheat sheep zones because I have no access to the available climatic data and the monthly livelihood capital. In contrast, I focus on the methodology of the research design to answer the questions “Who (some people) are concerned about how to adapt to the vulnerability of what to what in some place (where) at some time (when)?” I also emphasized on the socioeconomic adaptation that may cause the long-term lagged effects. For example, heterogeneous regions constraints determined different adaptive strategies, which means the next-year yield are affected by the decision of production according to today’s response. Such decision processes include the lag-influence in crop planting. Therefore, I considered the lagged effect of the drought on annual crop yield.

## Conclusions

Who (some people) are concerned about how to adapt to the vulnerability of what to what in some place (where) at some time (when)? It means the adaptation pathway should transit and transform integration into typology among environmental types (i.e., types of time, space, who) and vulnerability types (i.e., exposure, sensitivity, and adaptive capacity). I illustrated the hypothesis that the process of assessing vulnerability and selecting adaptations can be enhanced by using a combination of integration, optimization and typology based on heterogeneities in space, time, social factors, exposure, sensitivity and adaptive capacity, using the case of Australia’s wheat sheep zones over the period 1981–1999. Our analysis framework of integration and typology of vulnerability can be of benefit to policy makers in determining appropriate adaptive strategies, and for crop–climate researchers to integrate socioeconomic factors into crop models. Although there are many advantages to the so called “six types” framework, the complexity due to the many types and factors and their interaction represents one of its disadvantages. This provides an opportunity for further research. In the case of Australia in particular, further work would be useful to select more socioeconomic indicators, such as human capital and develop more sub-classifications, such as regions of good harvest in drought and crop failure regions without drought, which I leave for further work.

## Additional Information

**How to cite this article**: Huai, J. Integrations and Typologies of Vulnerability to Climate Change: A Case Study from Australian Wheat Sheep Zones. *Sci. Rep*. **6**, 33744; doi: 10.1038/srep33744 (2016).

## Supplementary Material

Supplementary Information

## Figures and Tables

**Table 1:** Optimal vulnerability index correlates with seven financial categories using Spearman’s nonparametric method.

Categories (the first principal component)	*VI*^11^						*VI*^12^						Expected signs
	wheat		barley		oats		wheat		barley		oats		
	R	S	R	S	R	S	R	S	R	S	R	S	
Capital													−
Op. capital - land & improvements ($)							−0.295*	−0.499**					−
Op. capital - total ($)							−0.324**	−0.492**					−
Average capital - total ($)							−0.280*	−0.473**					−
Cl. capital - total ($)							−0.285*	−0.453**					−
Op. capital - other stocks ($)	−0.217	−0.278	−0.101	−0.355			−0.206	−0.371**					−
Receipts													−
Receipts - off farm contracts ($)							0.086	−0.419**					−
Costs													−
Total services ($)			−0.1	−0.442*			−0.291*	−0.482**					−
Administration ($)	−0.202	−0.566**					−0.274*	−0.466**					−
Fertiliser ($)	−0.236	−0.298	−0.08	−0.187			−0.199	−0.414**					−
Fuel, oil & grease ($)							−0.245*	−0.326*					−
Total materials costs ($)			−0.142	−0.470**			−0.247*	−0.318*					−
Freight ($)			−0.091	−0.390*			−0.313*	−0.371**					−
Handling & marketing expenses ($)	−0.303*	−0.361*	−0.161	−0.305									−
Crop & pasture chemicals ($)	−0.288*	−0.285	−0.15	−0.149			−0.296*	−0.423**					−
Debt													−
Opening debt - total ($)	−0.284*	−0.385*					−0.393**	−0.472**					−
Closing debt - banks include State, CDB ($)	−0.229	−0.386*					−0.344**	−0.422**					−
Closing debt - working capital ($)	−0.166	−0.495**					−0.267*	−0.400**					−
Closing debt - total ($)	−0.211	−0.372*					−0.295*	−0.388**					−
Closing debt - land purchase ($)	−0.24	−0.13					−0.288*	−0.264*	−0.441**	−0.234			−
Closing debt - plant & livestock ($)			−0.183	−0.374*			−0.1	−0.199	−0.273*	−0.289			−
Family farm measures													−
Family farm income ($)					0.077	−0.201					0.039	−0.291*	−
Farm performance													−
Farm cash income ($)					0.054	−0.382*			0.177	−0.122	0.061	−0.24	−
Profit at full equity ($)					0.01	−0.285					−0.068	−0.286*	−
Other													+
Farm equity ratio at 30 June (%)	0.236	0.202					0.262*	0.148					+
Farm liquid assets ($)	−0.234	−0.481**	−0.290*	−0.512**									−

^*^Correlation is significant at the 0.05 level (2-tailed).

^**^Correlation is significant at the 0.01 level (2-tailed).

**Table 2:** Droughts lagged by one year have a clear negative effect on crop yield.

Duration	Lagged–one year						Same year					
Exposure	Drought index				Wet index		Drought index				Wet index	
Index	SNR	WSNR	SNRM	WSNRM	MSNR	MWSNR	SNR	WSNR	SNRM	WSNRM	MSNR	MWSNR
Wheat yield	−0.520**	−0.501**	−0.510**	−0.489**	0.505**	0.474**	−0.212**	−0.201**	−0.204**	−0.212**	0.157*	0.156*
Barley yield	−0.422**	−0.402**	−0.412**	−0.391**	0.453**	0.426**	−0.137*	−0.11	−0.12	−0.135*	0.09	0.1
Oats yield	−0.246**	−0.236**	−0.232**	−0.222**	0.314**	0.300**	−0.166*	−0.165*	−0.167*	−0.164*	0.193*	0.200**
Wheat failure	0.305**	0.289**	0.315**	0.291**	−0.250**	−0.247**	0.12	0.11	0.11	0.12	−0.09	−0.07
Barley failure	0.248**	0.227**	0.260**	0.234**	−0.205**	−0.208**	0.05	0.04	0.04	0.05	0	0
Oats failure	0.13	0.12	0.141*	0.13	−0.160*	−0.190*	0.147*	0.136*	0.133*	0.148*	−0.15	−0.13

^*^Correlation is significant at the 0.05 level (2-tailed).

^**^Correlation is significant at the 0.01 level (2-tailed).

**Table 3:** Typology of vulnerability was established by analysis of debt and cost, capital, receipts, assets, family and farm performance.

Types	Financial constraints		Resilient region codes			Sensitive region codes			Adaptive strategies
			wheat	barley	oats	wheat	barley	oats	
			422	121	121	122	122	221	
			421	322	321	223	223	223	
			522	522	521	321	321	421	
			521	422	522	221	221	123	
**1**	**Capital**	221,222,223,422	422	422		221,223	221,223	221,223	Incentivize farmers to acquire additional plant & equipment and reduce opening capital.
**2**	**Farm assets**								Accumulate farm equity and use the farm’s liquid assets more efficiently.
**3**	**Total receipts**	122,223,123,222				122,223	122,223	223,123	Protect agricultural receipts from transforming into off-farm industrial activity and avoid substitutes between crops.
**4**	**Farm performance**	122,223,321			321	122,223,321	122,223,321	223	Control the risk of adding farm cash income and profit at full equity through market mechanism, such as taking out a kind of insurance, through which farmers pay a small amount of cost to gain huge compensation for the huge economic loss due to future uncertainties.
**5**	**Family farm**								Improve family farm performance but protect income from transforming into off-farm industrial activities.
**6**	**Cost**	121,322		121,322	121				Reduce the cost of seed, fodder, fertiliser, crop & pasture chemicals, administration, rates and interest paid. Increase purchases of beef cattle, technological investments, capital investment, saving, etc.
**7**	**Total debt**	121,322,521,522	521,522	121,322,522	121,521,522				Reduce the risk of total debt, financial debt and land purchasing

The corresponding adaptive strategies for each type of vulnerability were determined by the financial characteristics of vulnerable cases for wheat, oats and barley. The numbers represent region codes.
