# Corrigendum: Integration and Typologies of Vulnerability to Climate Change: A Case Study from Australian Wheat Sheep Zones

**DOI:** 10.1038/srep40456

**Published:** 2017-01-12

**Authors:** Jianjun Huai

Scientific Reports 6: Article number: 3374410.1038/srep33744; published online: 09 27 2016; updated: 01 12 2017

This Article contains typographical errors in Equation (1).


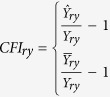


should read:


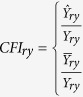


